# Supplementary material for: Urinary C-Peptide to Creatinine Ratio (UCPCR) as Indicator for Metabolic Risk in Apparently Healthy Adults—A BioPersMed Cohort Study
Source: Nutrients. 2023 Apr 25;15(9):2073. doi: 10.3390/nu15092073 (PMC10181310; doi:10.3390/nu15092073)
Supplement: Supplementary file 1 [file nutrients-15-02073-s001.zip › nutrients-2341463-supplementary.pdf]

nutrients

Supplementary data

Table S1. Baseline characteristics of the study group using propensity-matched data. Values are represented as median ± interquartile range (IQR, 25–75th percentile). NA means not applicable due to non-statistically significant values from Kruskal–Wallis test. Laboratory-based reference ranges are described in parentheses at the appropriate parameters. BMI: body mass index; WHR: waist-to-hip ratio; GGT: gamma-glutamyl transferase; AUC: area under the curve; HbA1c: hemoglobin A1c; HOMA-IR: Homeostatic Model Assessment for Insulin Resistance; ISI: Insulin Sensitivity Index; UCP: urinary *C*-peptide; UCR: urinary creatinine; UCPCR: urinary *C*-peptide-to-creatinine ratio.

Variables	All Groups (n = 217)	Healthy (n = 87)	PreDM (n = 87)	DM N = 43	*p*-Value	Healthy vs PreDM (*p*-adj.)	Healthy vs DM (*p*-adj.)	PreDM vs DM (*p*-adj.)
**Age (years)**	61 (53.50–66.50)	61 (53–67)	59 (53–66)	61 (58–67)	0.439	NA	NA	NA
**BMI (kg/m²)**	28.07 (25.50 (31.31)	27.25 (25–30.20)	27.80 (25.60–30.80)	30.80 (26.10–33.90)	**0.003**	1.000	**0.002**	**0.021**
**Hip (cm)**	103 (97–109.50)	103 (96–108)	103 (95–109)	108 (102–118)	**0.001**	1.000	**0.002**	**0.003**
**Waist (cm)**	98 (91–106.50)	98(88–105)	96 (90–105)	107 (97–116) **NS**	**<0.001**	1.000	**0.001**	**0.001**
**WHR**	0.96 (0.91–1.00)	0.95 (0.90–0.99)	0.96 (0.90–1.01)	0.98 (0.92–1.02)	0.221	NA	NA	NA
**Weight (kg)**	83 (75–93.50)	83 (75–94)	82 (74–88)	88 (80–103)	**0.027**	1.000	0.101	**0.025**
**Height (cm)**	173 (167–178)	174 (168–179)	173 (165–179)	172 (165–178)	0.534	NA	NA	NA
**Total lean mass (kg)**	54.35 (44.27–61.08)	55.08 (44.85–61.73)	52.92 (43.77–57.93)	56.78 (45.29–62.98)	0.205	NA	NA	NA
**Total fat mass (kg)**	28.81 (22.67–35.23)	27.21 (22.65–33.30)	28.26(22.15–36.25)	31.33 (27.07–37.66)	**0.014**	0.991	**0.011**	0.102
**GGT (U/L) (Female: 38 U/L Male: 55 U/L)**	29 (19–48.50)	24 (17–44)	31 (21–49)	45 (23–86)	**<0.001**	0.106	**0.001**	0.141
**Fasting blood glucose (mg/dL) (70–100 mg/dL)**	100 (92–110)	91 (87–95)	104 (100–108)	140 (123–164)	**<0.001**	**<0.001**	**<0.001**	**<0.001**
**AUC glucose (mg/dL per time unit)**	17,745 (14,280–21,892.50)	14,010 (12,765–15,840)	18,795 (16,815–20,730)	28,110 (24,660–34,470)	**<0.001**	**<0.001**	**<0.001**	**<0.001**
**HbA1c (mmol/mol) (20–42 mmol/mol)**	38 (36–42)	37 (35–38)	39 (37–42)	52 (44–59)	**<0.001**	**<0.001**	**<0.001**	**<0.001**
**Fasting blood *C*-peptide (ng/mL) (0.78–1.89 ng/mL)**	1.76(1.30–2.48)	1.38 (1.10–1.85)	1.98 (1.44–2.58)	2.55 (1.77–3.35)	**<0.001**	**<0.001**	**<0.001**	**0.023**
**1-h stimulated blood *C*-peptide (ng/mL)**	7.27 (5.56–9.31)	7.22 (5.18–9.10)	7.82 (6.20–10.34)	6.01 (4.54–8.15)	**0.001**	**0.049**	0.354	**0.001**
**2-h stimulated blood *C*-peptide (ng/mL)**	7.20 (4.84–9.61)	5.71 (4.13–8.18)	7.94 (5.74–11.07)	8.14 (6.09–9.75)	**<0.001**	**<0.001**	**0.007**	1.000

**AUC *C*-peptide**	705.30 (551.48–889.35)	650.10 (507.75–823.65)	786.90 (619.65–961.95)	686.40 (529.35–874.95)	**0.005**	**0.005**	1.000	0.114
**Fasting blood insulin (mU/L) (3–25 mU/L)**	11.60 (8.40–18.15)	10 (7.30–13.70)	11.90 (9.20–19.00)	16.10 (10.10–22.70)	**<0.001**	**0.006**	**<0.001**	0.516
**AUC insulin**	6781.50 (4551–11,465.25)	6736.50 (4426.50–10,651)	7872 (4587–14,185.50)	6274.50 (3961.50–9388.50)	0.351	NA	NA	NA
**HOMA-IR**	2.94 (2.02–4.80)	2.17 (1.55–3.24)	3.09 (2.28–4.81)	5.07 (3.60–9.22)	**<0.001**	**<0.001**	**<0.001**	**0.002**
**HOMA-beta (%)**	113.89 (76.83–174.86)	132.48 (98–187.20)	109.13(84.65–178.05)	75.27 (40.91–115.41)	**<0.001**	0.451	**<0.001**	**0.006**
**ISI Stumvoll**	0.08 (0.04–0.09)	0.08 (0.06–0.10)	0.08 (0.01–0.09)	0.06 (0.04–0.09)	**0.005**	0.056	**0.008**	0.811
**ISI Cederholm**	37.40 (25.27–52.25)	50.28 (37.43–59.48)	34.87 (25.27–47.31)	20.28 (15.58–26.81)	**<0.001**	**<0.001**	**<0.001**	**<0.001**
**Matsuda index**	3.60 (1.99–5.32)	4.40 (3.21–6.99)	3.09 (1.72–4.88)	2.18 (1.28–3.35)	**<0.001**	**0.001**	**<0.001**	**0.014**
**Blood creatinine (mg/dL) (Female: 0.50-0.90 mg/dL Male: 0.70-1.20 mg/dL)**	0.91 (0.80–1.03)	0.91 (0.78–1.03)	0.93 (0.83–1.05)	0.86 (0.76–0.96)	0.163	NA	NA	NA
**UCP (nmol/L)**	8.08 (4.32–11.84)	6.09 (3.24–10.23)	8.24(4.73–11.85)	10.83 (8.31–15.43)	**<0.001**	**0.043**	**<0.001**	**0.031**
**UCR (mmol/L)**	11.93 (7.78–16.44)	12.38 (7.69–17.33)	11.85 (7.69–16.00)	11.93 (8.49–16.09)	0.494	NA	NA	NA
**UCPCR (nmol/mmol)**	0.67 (0.41–1.02)	0.48 (0.35–0.79)	0.76 (0.46–1.10)	0.92 (0.59–1.80)	**<0.001**	**0.002**	**<0.001**	0.214
